# Supplementary material for: A description of the genus Denitromonas nom. rev.: Denitromonas iodatirespirans sp. nov., a novel iodate-reducing bacterium, and two novel perchlorate-reducing bacteria, Denitromonas halophila and Denitromonas ohlonensis, isolated from San Francisco Bay intertidal mudflats
Source: Microbiol Spectr. 2023 Sep 29;11(5):e00915-23. doi: 10.1128/spectrum.00915-23 (PMC10581121; doi:10.1128/spectrum.00915-23)
Supplement: Supplemental material legends — Supplemental table titles. [file spectrum.00915-23-s0001.pdf]

Supplemental Material

Table S1: List of *Denitromonas* spp. 16S sequences

Table S2: Protein subfamilies of all genomes searched

Table S3: Protein subfamilies unique to *Denitromonas* spp.

Table S4: Protein subfamilies unique to close relatives of *Denitromonas* spp.

Table S5: Protein subfamilies missing from *Denitromonas* spp.

Table S6: Average nucleotide identities presented as percent pairwise identity

Table S7: Jaccard similarity index scores
